# Supplementary material for: A Preliminary Study on the Effect of Adding Sugarcane Syrup on the Flavor of Barley Lager Fermentation
Source: Foods. 2024 Jul 25;13(15):2339. doi: 10.3390/foods13152339 (PMC11311557; doi:10.3390/foods13152339)
Supplement: Supplementary file 1 [file foods-13-02339-s001.zip › foods-3102848-supplementary.pdf]

foods

Supplementary Materials

Physico-chemical parameters, volatile compounds content, parameters and flavor stability of lager beer by addition of sugarcane syrup

Hechao Lva,[1], Yusheng Jia[1], Chaoyi Liu[1], Jia Xua[1], Caifeng Xie[1,2,3] Kai Li[1,2,3], Fangxue Hang[1,2,3,*],

[1] College of Light Industry and Food Engineering, Guangxi University, Nanning 530004, China
[2] Provincial and Ministerial Collaborative Innovation Center for Sugar Industry, Nanning 530004, China
[3] Engineering Research Center for Sugar Industry and Comprehensive Utilization, Ministry of Education, Nanning 530004, China
* Correspondence: hangfx@163.com (F.H.)

1. Refer to the patent of the People's Republic of China for the specific fermentation process of this product: CN 114164068 A.

The specific fermentation process can be summarized as follows:

Wort Preparation: 97% of barley malt, and 3% of caramel malt are crushed with a crusher until the hulls are broken. Proteins are left to stand for 30 minutes, followed by saccharification, a 70°C iodine test until the wort does not change color under iodine (blue-violet color is not altered), insulation, and filtration.

Yeast activation: take 20 g of dry yeast, put it into 200 mL of sterilized water, let it stand for 20 min, shake it well, incubate it at a constant temperature of 32 °C for 5 min, add 200 mL of 12 °C wort and incubate it for 30 min, shake it well continuously, and the activation is finished, and then it can be put into the feed (the activation solution is 9×10^9 cells/mL). Addition: the amount of yeast added is 1%, and the initial concentration of medium is 9×10^7 cells/mL. The initial concentration of the medium is 9×10^7 cells/mL.

Fermentation: 15 days at 12°C. Sugar syrup: Sugar syrup (concentrated syrup) is diluted with boiled and cooled domestic drinking water to the same brix as the wort and is added to the brewing tank in a specific ratio of sucrose syrup to wort, with a pH value ranging from 5.13 to 5.50. Fermentation is complete when the brix has dropped by 0.2 °P compared to the previous day.The liquid is filtered and sterilized; it is heated to 60°C for 20 minutes, then sterilized and canned. The finished beer is then available.

2. Sugarcane syrup production method: Unlike traditional syrups made by chemical methods,the sugarcane juice is pressed into the 50nm membrane, the filtrate after the membrane is evaporated and concentrated to 60 -65 brix.

Table S1. Sources of production of commercially available beer

Lager beer	Manufacturer
CAL_1	Beijing Yanjing Brewery Co.
CAL_2	Shandong Tsingtao Brewery Co.

Table S2. Sources of raw materials for beer brewing

materials	Manufacturer
barley malt	Jinan Shuangmai Beer Raw Material Co.

caramelized malt	Jinan Shuangmai Beer Raw Material Co.
lager yeast	Angie's Yeast Co.
hops	Palodin Imported Beer Ingredients, Inc.
sugarcane syrup	Guangxi Baiguitang Foodstuffs Co.

Table S3. Sensory evaluation table of sugarcane beer

Program	standard	point
color and luster(15 points)	Amber yellow in color with a bright, glossy finish	8～15
	Yellowish, average color, slight loss of light	5～7
	Yellowish, dull color, loss of light	0～4
beer foam (15 points)	High, creamy, white, long-lasting foam.	10～15
	Low foam height, coarse and not long lasting	6～9
	Poor foam retention	0～5
aroma(20 points)	Aromas of hops and sweet fruit, harmonized aromas, no aging.	15～20
	Fruity and hoppy aromas are more harmonized.	8～14
	Fruity and hoppy aromas not harmonized, overly fruity or boozy, rancid taste	0～7
taste(40 points)	Strong, full-bodied beer flavor, pure taste, strong mouthfeel, no off-flavors	30～40
	Average mouthfeel power, no odor	15～29
	Poor mouthfeel power, bad odor	0～14
clarification(10 points)	Clarity and transparency, no suspension	8～10
	Slightly suspended	4～7
	Visible suspended solids	0～3
